# A Comparison of Short- and Long-Term Therapeutic Outcomes of Infliximab- versus Tacrolimus-Based Strategies for Steroid-Refractory Ulcerative Colitis 

**DOI:** 10.1155/2016/3162595

**Published:** 2016-01-20

**Authors:** Katsuya Endo, Motoyuki Onodera, Hisashi Shiga, Masatake Kuroha, Tomoya Kimura, Keiichiro Hiramoto, Yoichi Kakuta, Yoshitaka Kinouchi, Tooru Shimosegawa

**Affiliations:** Division of Gastroenterology, Tohoku University Graduate School of Medicine, 1-1 Seiryo, Aoba-ku, Sendai 980-8574, Japan

## Abstract

*Background/Aims*. Antitumor necrosis factor antibodies and calcineurin inhibitors have shown good therapeutic efficacy for steroid-refractory ulcerative colitis (UC). Although some studies have compared the efficacy of infliximab (IFX) and cyclosporin A, there are no published studies comparing IFX and tacrolimus (Tac). This study aimed to compare therapeutic efficacies between IFX- and Tac-based strategies for steroid-refractory UC.* Methods*. Between July 2009 and August 2013, 95 patients with steroid-refractory UC received either IFX (*n* = 48) or Tac (*n* = 47) in our hospital. In the IFX group, the patients continued to receive maintenance treatment with IFX. In the Tac group, patients discontinued Tac treatment up to 3 months and subsequently received thiopurine. We retrospectively compared the therapeutic outcomes between the groups.* Results*. There was no significant difference in the colectomy-free rate, clinical remission rate, and clinical response rate at 2 months between the groups. However, relapse-free survival was significantly higher in the IFX group than in the Tac group (*p* < 0.001; log-rank test). The proportions of serious adverse events did not differ between the groups.* Conclusion.* The findings of our study showed that IFX and Tac have similar short-term therapeutic efficacy for steroid-refractory UC. Maintenance treatment with IFX, however, yields better long-term outcomes than Tac-thiopurine bridging treatment.

## 1. Introduction

Ulcerative colitis (UC) is a chronic relapsing inflammatory bowel disease of unknown etiology. 5-Aminosalicylates or topical treatments are usually the first-line treatment, and corticosteroids are the second-line treatment. Owing to the excellent efficacy of systemic administration of corticosteroids, most patients achieve remission even if they required second-line treatment. However, 20–40% of patients fail to respond to corticosteroids (steroid-refractory UC) or fail to maintain remission without them (steroid-dependent UC) [1–5]. Previously, most patients with steroid-refractory or steroid-dependent UC would avoid total colectomy. However, third-line salvage therapies for steroid-refractory cases, such as thiopurines [6–8], antitumor necrosis factor (TNF) antibodies [9–12], and calcineurin inhibitors [13–16], have been developed over the recent two decades.

Thiopurines such as azathioprine and 6-mercaptopurine are commonly used to maintain remission and are also suitable for reducing the dose of corticosteroids in patients with steroid-dependent UC. On the other hand, anti-TNF antibodies, such as infliximab (IFX) or adalimumab, and calcineurin inhibitors, such as cyclosporin A (CsA) or tacrolimus (Tac), have shown good salvage therapeutic efficacies for inducing remission in steroid-refractory UC.

Anti-TNF antibodies are usually administered for both inducing and maintaining remission consecutively. However, calcineurin inhibitors are used mainly for inducing remission. Other drugs such as thiopurines are used for maintaining remission when the remission was achieved with calcineurin inhibitors because of the absence of long-term treatment data. To understand the difference in efficacy between treatment with anti-TNF antibodies and calcineurin inhibitors, some studies have compared the therapeutic outcomes with IFX-based treatment versus CsA-based treatment [17–20]. However, whether IFX or CsA should be chosen to treat patients with steroid-refractory UC remains controversial. Moreover, there are no published data with which to compare therapeutic efficacies between IFX-based treatment and Tac-based treatment.

Tac is a newly developed calcineurin inhibitor that inhibits the transcription of interleukin-2 and interferon-gamma in T lymphocytes, similar to the mechanism of CsA. The utility of Tac for treating refractory UC has been reported in some studies: the short-term response rates range from 55 to 98%, with less severe adverse events than with CsA [15, 16, 21–24]. Therefore, Tac is now strongly regarded as one of the main therapeutic options when treating steroid-refractory UC. However, whether long-term maintenance therapy using Tac is effective and safe remains unclear. In Japan, the duration of Tac administration is officially limited to up to 3 months by the Ministry of Health because of the absence of long-term data regarding the efficacy and safety of this regimen. Therefore, in the current situation, Tac can only be used to induce remission in steroid-refractory UC and as a rescue and bridging drug before initiating treatment with thiopurines [24]. Although the evidence concerning the efficacy of Tac in inducing remission seems to be adequate, there are only a few reports describing the long-term outcomes of the Tac-thiopurine bridging strategy. Furthermore, there also have been no published studies comparing the short- and long-term efficacy of IFX-based treatment and Tac-based treatment for steroid-refractory UC.

In order to make an appropriate therapeutic choice for steroid-refractory UC, it is important to make evidence comparing the outcomes between an IFX-based strategy and a Tac-based strategy. Therefore, the aim of this study was to compare the short- and long-term efficacy of IFX-based strategy and Tac-thiopurine bridging strategy for the treatment of steroid-refractory UC. We retrospectively reviewed data regarding the clinical courses of patients with steroid-refractory UC in our hospital who were treated with either IFX or Tac and then evaluated the comparable therapeutic outcomes in both groups. To the best of our knowledge, this is the first report to compare the short- and long-term effectiveness and safety of IFX-based treatment and Tac-based treatment for steroid-refractory UC.

## 2. Patients and Methods

### 2.1. Patients

Between July 2009 and August 2013, a total of 95 patients with corticosteroid-refractory UC received either IFX or Tac in our hospital. All consecutive 95 patients were included in this study. Of those, 48 patients received IFX (IFX group) and 47 patients received Tac (Tac group), respectively. In all cases, the diagnosis of UC and disease activity was confirmed according to standardized criteria by prior clinical assessment, radiology, endoscopy, and histology. As for the definition of the response to corticosteroids, UC was regarded as steroid-refractory if the patient was in either steroid-refractory or steroid-dependent state defined as follows. Steroid-refractory state was defined as lack of a meaningful clinical response to oral or intravenous prednisolone more than 30 mg/day over at least two weeks. Steroid-dependent state was defined as occurring if prednisolone cannot be tapered to less than 10 mg/day without recurrent disease or if relapse occurs within three months of stopping prednisolone.

We retrospectively reviewed the medical records of all 95 patients and compared the short- and long-term therapeutic efficacy between the IFX group and Tac group. All patients provided their informed consent for the present study.

### 2.2. Treatment Strategy

Criteria for choosing IFX or Tac depended on the treatment policy in our hospital. The important factor is current situation of thiopurine administration. Basically, IFX is mainly chosen for thiopurine-refractory or thiopurine-intolerant patient, and Tac is mainly chosen for thiopurine-naïve patient. Most of the patients enrolled in this study were treated under this treatment strategy. However, not all the patients followed this strategy. We selected another treatment arm when we could not obtain the informed consent from the patients or the patients had complications at baseline that could possibly get worse, such as renal dysfunction in Tac-based strategy.

Patients in the IFX group were treated with a 5 mg/kg infusion at weeks 0, 2, and 6. When clinical response was observed, maintenance treatment with IFX (5 mg/kg every 8 weeks) was consecutively initiated. Concomitantly, thiopurines were administered if the patients tolerated the initial regimen well. The dose of thiopurines was adjusted to achieve white blood cell counts between 3000 and 5000 cells/*μ*L in a manner similar to that previously reported [25]. Generally, maintenance treatment with IFX was continued unless clinical relapse occurred. However, in a few cases in which both clinical and endoscopic remissions were achieved, IFX treatment was subsequently discontinued according to the physician's decision.

Patients in the Tac group were treated with adjusted oral doses of Tac. When starting Tac treatment, inpatient therapy was the standard approach in our hospital. The initial dose of Tac was 0.1 mg/kg per day twice daily. Blood was collected to determine the tacrolimus whole-blood trough concentrations at 12 hours, 24 hours, 2–5 days, and 7–10 days and every 7 days after providing the initial dose during the early period of therapy. The dosage was then adapted to achieve a trough level between 10 and 15 ng/mL to induce remission. The equation (previous dose × 12.5 mg/mL/the blood trough level) was used for the dose adjustment during the induction periods. After inducing clinical remission, Tac trough concentrations were maintained at a lower level, between 5 and 10 ng/mL. The equation (previous dose × 7.5 mg/mL/the blood trough level) was used for the dose adjustment during the maintenance periods. The patients were hospitalized until their clinical conditions had stabilized and their tacrolimus levels were within the therapeutic range. Thereafter, the patients were regularly seen as outpatients at least every four weeks.

Thiopurines were also administered to the thiopurine-naïve patients within the first 4 weeks of Tac induction if the patients tolerated the regimen. The patients using thiopurine at the start of Tac therapy concomitantly continued to take them. The thiopurine dose was adjusted to achieve white blood cell counts between 3000 and 5000 cells/*μ*L in a manner similar to that previously reported [25]. Maintenance treatment using Tac was generally withdrawn at 3 months after the first administration if clinical remission was achieved; subsequently, the patients were bridged to thiopurine monotherapy if they tolerated the regimen. In a few cases of failure to achieve clinical remissions at 3 months after the first administration, the patients continued to receive Tac beyond the limit of 3 months. The Tac-thiopurine bridging strategy described above was based on the official limitation of the Tac administration period for UC by the Ministry of Health in Japan.

### 2.3. Analysis of Therapeutic Efficacy

The short-term therapeutic efficacy was evaluated at 2 months after starting each treatment. In this study, we evaluated the patients' clinical disease activity using the Rachmilewitz Clinical Activity Index (CAI) [26]. Clinical remission was defined as an estimated CAI score of 4 or less, and clinical response was defined as a reduction in CAI score by more than 1 point. We evaluated the proportion of patients who achieved clinical remission and clinical response at 2 months. We also evaluated the proportion of colectomy-free patients at 2 months. To assess for long-term therapeutic efficacy, we evaluated the relapse-free survivals and colectomy-free survivals in both groups. Relapse-free survival was defined as no need for salvage treatment for remission. We also assessed the adverse events in the IFX group and Tac group. Renal dysfunction was defined as an increase in serum creatinine levels to >30% above the baseline level, according to a previous study [15]. A serious adverse event was defined as that which causes drug withdrawal.

### 2.4. Statistical Analysis

Proportions between two groups were compared by using the Fisher exact probability test, and continuous variables were compared with the Mann-Whitney *U* test. Kaplan-Meier plots and log-rank tests were performed to analyze relapse-free survival in the two groups. A Cox proportional hazard model was used to identify the risk factors for relapse in the two groups. A *p* value < 0.05 was regarded as statistically significant for between-group comparisons. All statistical analyses were performed using JMP Pro 11.0.0. (SAS Institute Inc., Cary, North Carolina, USA).

## 3. Results

### 3.1. Baseline Characteristics

A total of 95 patients were analyzed in this study: 48 in the IFX group and 47 in the Tac group. Baseline patients' characteristics are shown in Table 1. No differences were found in the epidemiologic characteristics (age at treatment onset and sex) between the two groups, except for disease duration. The proportion of thiopurine-naïve patients at treatment onset was higher in the Tac group than in the IFX group (79% versus 46%, *p* = 0.0004). In the IFX group, 14 out of 48 patients had previous Tac treatment history. In these 14 patients, 6 patients were Tac-refractory and 8 patients were the relapse cases after withdrawal of Tac. In the Tac group, 5 out of 47 patients had previous IFX treatment history. In these 5 patients, 4 patients were IFX-refractory and 1 case was IFX-intolerant.

The patient with inactive colitis was not included in the study. However, some patients with mild clinical activity were included. Precisely, in the IFX group, 2 patients with CAI 2 and 1 patient with CAI 4 were included. In the Tac group, 3 patients with CAI 4 were included. However, all 6 patients were steroid-refractory cases and had endoscopic active colitis (moderate activity). According to the laboratory parameters (hemoglobin, albumin, and C-reactive protein) and CAI score at treatment onset, the Tac group had higher disease severity than the IFX group.

The median follow-up period was 24.5 months (range, 2.7–50 months) in the IFX group and 5.8 months (range, 0.6–52 months) in the Tac group.

### 3.2. Overall Treatment Outcomes

**Figure 1:**
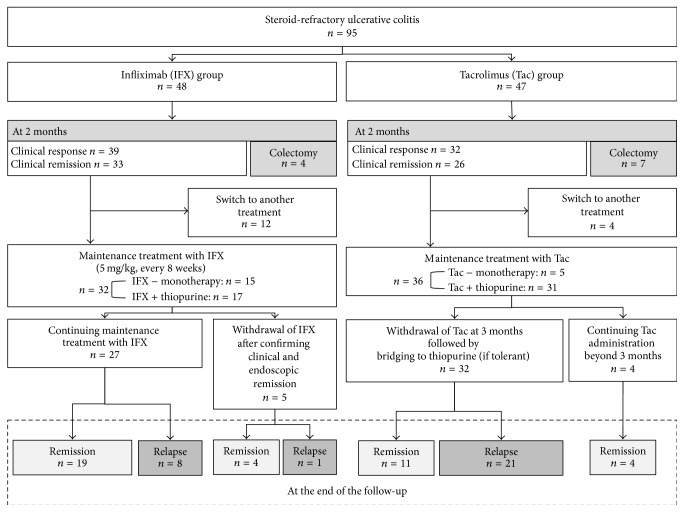
Flowchart of treatment outcomes in the infliximab group (*n* = 48) and tacrolimus group (*n* = 47).

Treatment outcomes of all patients are shown as a flowchart in Figure 1. In the IFX group (*n* = 48), colectomy was performed in 4 patients within 2 months after starting IFX treatment. At 2 months, clinical response was achieved in 39 patients, and clinical remission was achieved in 33 patients, respectively. Maintenance treatments with IFX were administered in 32 patients. In these 32 patients, 17 (53.1%) were treated with a combination of IFX and thiopurine and 15 (46.9%) were treated with IFX monotherapy. The maintenance treatments with IFX were continued in 27 patients, of whom 8 experienced relapse during the observation periods. Endoscopic remission (defined as a Rachmilewitz Endoscopic Index of ≤4 [26]) was achieved at 2 months in 13 out of these 27 cases who received maintenance treatment with IFX. Relapse was observed in 3 out of 13 cases with endoscopic remission during the observation periods. In the 13 cases with endoscopic remission, 8 achieved mucosal healing (defined as a Rachmilewitz Endoscopic Index of 0) at 2 months. Relapse was observed in 2 out of 8 cases with mucosal healing. The maintenance treatments with IFX were withdrawn in 5 patients with clinical and endoscopic remission, of whom 1 experienced relapse.

In the Tac group (*n* = 47), the median days of achieving high trough level was 5 days (range: 1–20 days). Colectomy was performed in 7 patients within 2 months after starting Tac treatment. At 2 months, clinical response was achieved in 32 patients, and clinical remission was achieved in 26 patients, respectively. Maintenance treatments with Tac were administered in 36 patients. In these 36 patients, thiopurines were administered in 31 patients (86.1%); five patients (13.9%) were treated with Tac monotherapy because of intolerance to thiopurines. In 32 of these 36 patients, Tac administration was withdrawn at 3 months followed by bridging to thiopurine (if tolerant), and 21 patients experienced relapse during the observation periods. In these 32 cases, endoscopic evaluations were performed in 23 cases before stopping Tac. Endoscopic remission (defined as a Rachmilewitz Endoscopic Index of ≤4 [26]) was achieved in 18 cases. Relapse was observed in 12 out of 18 cases with endoscopic remission during the observation periods. In the 18 cases with endoscopic remission, 11 achieved mucosal healing (defined as a Rachmilewitz Endoscopic Index of 0) before stopping Tac. Relapse was observed in 9 out of 11 cases with mucosal healing. In 4 of 36 patients with primary responses, Tac administration was continued beyond 3 months, and no patient experienced relapse.

### 3.3. Short-Term Therapeutic Efficacy

**Figure 2:**
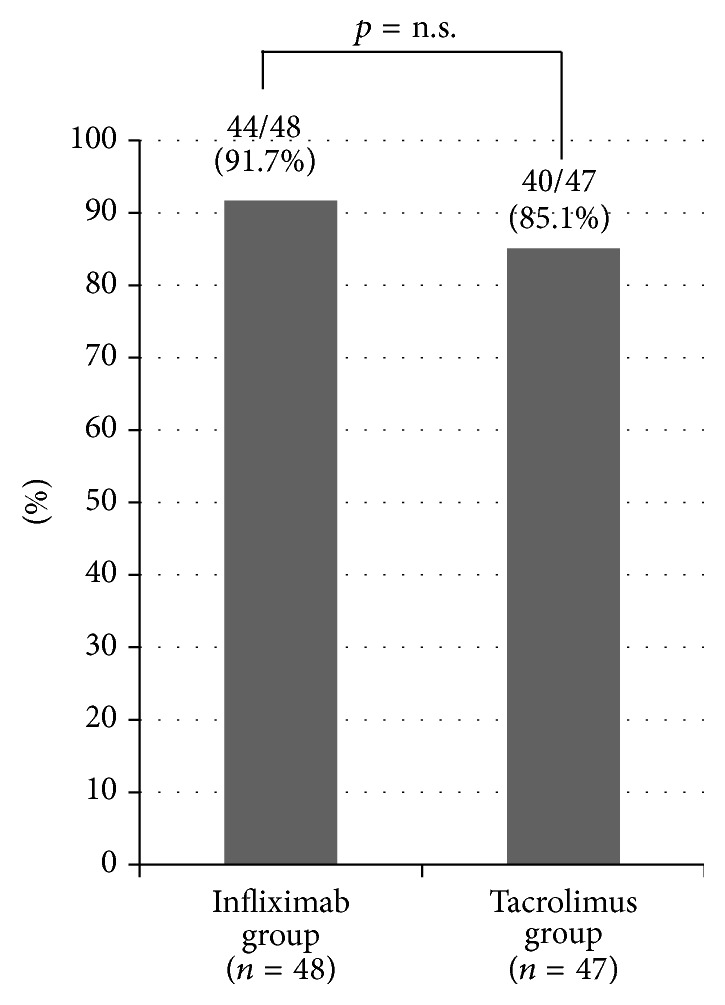
Colectomy-free rate at 2 months. Colectomy-free rate at 2 months was 91.7% in the infliximab group and 85.1% in the tacrolimus group, without a significant difference between the two groups.

**Figure 3:**
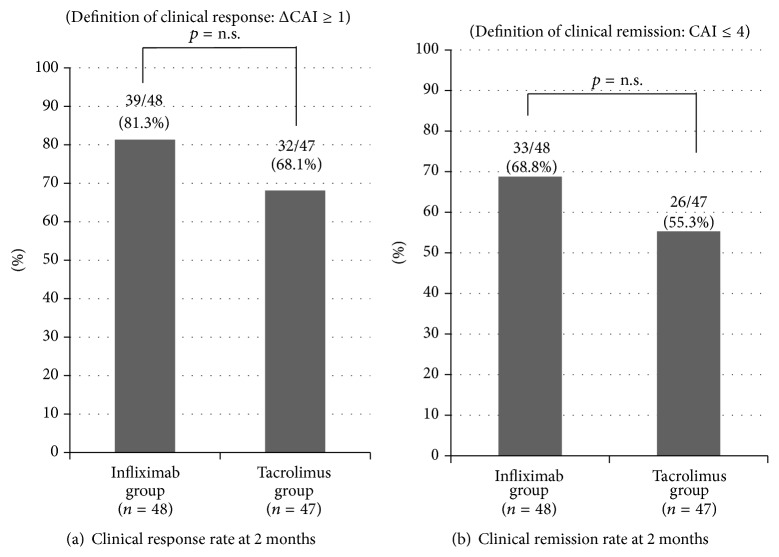
Clinical remission rate and clinical response rate at 2 months. (a) Clinical response rate at 2 months was 81.3% in the infliximab group and 68.1% in the tacrolimus group, without a significant difference between the two groups. (b) Clinical remission rate at 2 months was 68.8% in the infliximab group and 55.3% in the tacrolimus group, without a significant difference between the two groups.

The colectomy-free rate at 2 months was 91.7% in the IFX group and 85.1% in the Tac group (Figure 2). There was no significant difference between the two groups. The clinical response rate at 2 months was 81.3% in the IFX group and 68.1% in the Tac group (Figure 3(a)). There was no significant difference between the two groups. The clinical remission rate at 2 months was 68.8% in the IFX group and 55.3% in the Tac group (Figure 3(b)). There was no significant difference between the two groups. The clinical remission rate at 2 months in switching treatments (IFX therapy in patients previously treated with Tac or Tac therapy in those previously treated with IFX) was also evaluated. In the IFX group, 14 patients previously underwent treatment with Tac. Among these, 8 (57.1%) reached clinical remission at 2 months. In the Tac group, 5 patients previously underwent treatment with IFX, and none reached clinical remission at 2 months.

### 3.4. Long-Term Therapeutic Efficacy

**Figure 4:**
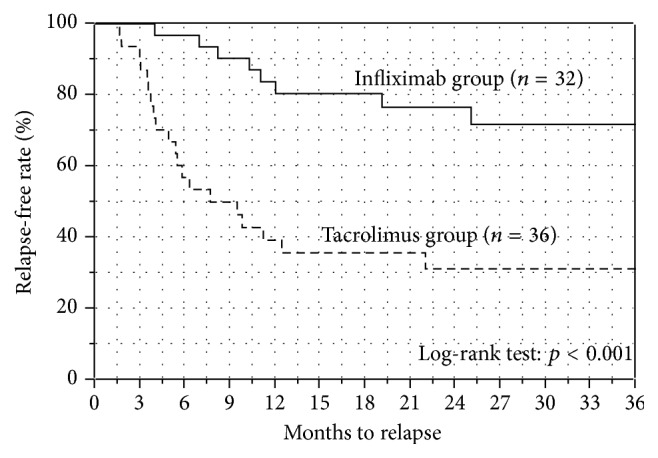
Kaplan-Meier plots for relapse-free survival. The relapse-free survival rate at 3, 6, 12, and 18 months was 100%, 97%, 83%, and 83% in the infliximab group (*n* = 32) and 90%, 57%, 37%, and 37% in the tacrolimus group (*n* = 36), respectively. The relapse-free survival rate was significantly higher in the infliximab group than in the tacrolimus group (*p* < 0.001; log-rank test).

**Figure 5:**
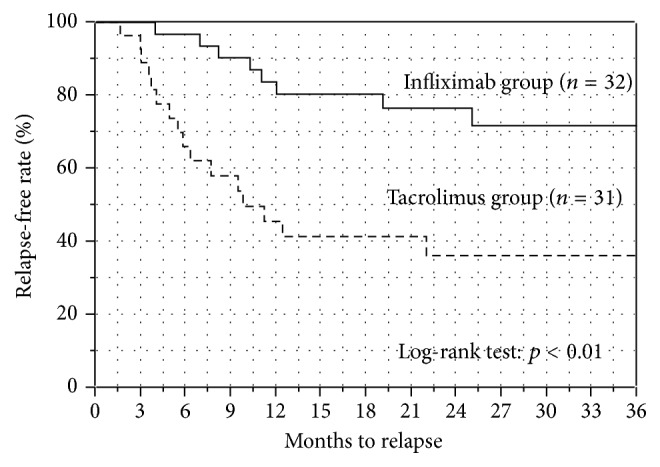
Kaplan-Meier plots for relapse-free survival (excluding 5 thiopurine-intolerant cases from the Tac group). Even though the 5 thiopurine-intolerant cases were excluded from the Tac group, the relapse-free survival rate was still significantly higher in the IFX group than in the Tac group (*p* < 0.01; log-rank test).

The Kaplan-Meier plots for relapse-free survival in both groups are shown in Figure 4. The relapse-free survival rate at 3, 6, 12, and 18 months was 100%, 97%, 83%, and 83% in the IFX group (*n* = 32) and 90%, 57%, 37%, and 37% in the Tac group (*n* = 36), respectively. The relapse-free survival rate was significantly higher in the IFX group than in the Tac group (*p* < 0.001; log-rank test). We also performed the Kaplan-Meier analysis and log-rank analysis excluding the thiopurine-intolerant cases in the Tac group (Figure 5). Even though the 5 thiopurine-intolerant cases were excluded from the Tac group, the relapse-free survival rate was still significantly higher in the IFX group than in the Tac group (*p* < 0.01; log-rank test).

Risk factors for relapse in long-term in each group were identified by using the Cox proportional hazard model. The result of the Cox model analysis for relapse in the IFX group is shown in Table 2. Disease duration at treatment was identified as an independent risk factor for relapse. The patients with a disease duration less than 5 years had a significantly higher risk for relapse (hazard ratio [HR], 9.17; 95% confidence interval [CI], 1.54–181.6; *p* = 0.012). Neither concomitant use of thiopurine nor withdrawal of IFX treatment was related to relapse.

The results of the Cox model for relapse in the Tac group are shown in Table 3. The absence of concomitant use of thiopurines and discontinuing Tac treatment beyond 3 months were identified as independent risk factors for relapse. The patients without concomitant thiopurine use had a significantly higher risk for relapse (HR, 6.83; 95% CI, 1.27–32.4; *p* = 0.027). The patients who did not continue to use Tac beyond 3 months had a significantly higher risk for relapse (HR, 8.33 × 108; 95% CI, 1.56–1.8 × 10290; *p* = 0.018).

**Figure 6:**
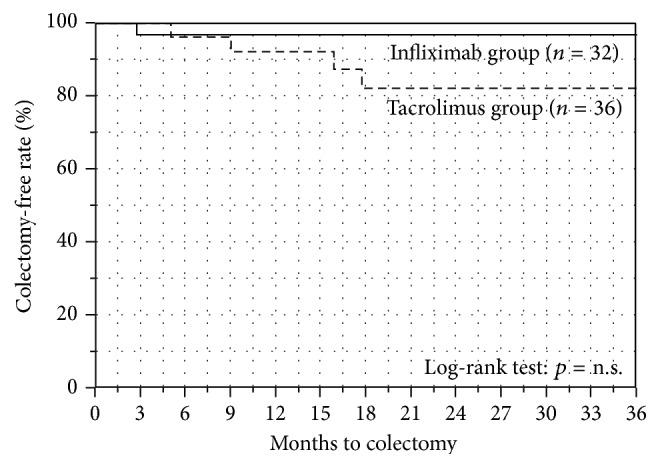
Kaplan-Meier plots for colectomy-free survival. The colectomy-free survival rate at 6, 12, and 18 months was 97%, 97%, and 97% in the IFX group (*n* = 32) and 96%, 92%, and 82% in the Tac group (*n* = 36), respectively. There was no significant difference between both groups.

The Kaplan-Meier plots for colectomy-free survival in both groups are shown in Figure 6. The colectomy-free survival rate at 6, 12, and 18 months was 97%, 97%, and 97% in the IFX group (*n* = 32) and 96%, 92%, and 82% in the Tac group (*n* = 36), respectively. There was no significant difference between both groups.

### 3.5. Adverse Events

The adverse events observed in both groups were shown in Table 4. In the Tac group, mild clinical symptoms such as hot flashes and tremors were frequently observed. However, the proportions of patients with serious adverse events, which caused drug withdrawal, were not significantly different between the two groups.

## 4. Discussion

In the present study, we evaluated the short- and long-term therapeutic efficacies of IFX-based strategy and Tac-based strategy for steroid-refractory UC. To the best of our knowledge, this is the first report comparing the efficacy between these strategies.

Firstly, our results showed that there were no significant differences in the colectomy-free rate, clinical remission rate, and clinical response rate at 2 months between the IFX group and the Tac group. These results indicated that IFX and Tac have similar short-term therapeutic efficacy for the treatment of steroid-refractory UC. When interpreting these results, we should pay special attention to the methods of Tac administration. In this study, we tightly adjusted the Tac dose to maintain the appropriate trough concentration. During the initial 2 weeks, the patients in the Tac group were treated by maintaining the high trough Tac concentration (10–15 ng/mL). A randomized controlled trial published by Ogata et al. showed that Tac has a trough concentration-dependent effect [15]; patients in the high trough concentration (10–15 ng/mL) group had better therapeutic outcomes than those in the low trough concentration (5–10 ng/mL) group. They proposed that the optimal target range appears to be 10–15 ng/mL in terms of efficacy for a 2-week regimen. We followed this proposal and treated the patients with the 2-week high trough strategy in the present study. Therefore, we conclude that Tac has similar short-term therapeutic efficacy compared with IFX as long as the dose is tightly adjusted sufficiently to maintain the high trough concentration during the 2 weeks.

In the present study, we also evaluated whether switching therapies (IFX therapy in patients previously treated with Tac or Tac therapy in those previously treated with IFX) has an adequate therapeutic efficacy or not. The results showed that clinical remission rate at 2 months was 57.1% in the IFX treatment in patients previously treated with Tac. On the other hand, none of the 5 patients previously treated with IFX reached the clinical remission at 2 months by Tac treatment. These results indicate that IFX therapy could be meaningful even in patients with Tac treatment failure. However, Tac therapy in patients with IFX treatment failure might not have adequate therapeutic efficacy. Because the number of patients who experienced a switching treatment in our study was quite small, further investigations are needed to clarify this issue.

Secondly, the results of the long-term outcomes in the present study showed remarkable differences between the IFX-based strategy and Tac-based strategy. The IFX-based strategy showed obviously better outcomes than the Tac-based strategy when comparing the relapse-free survival rates by using the Kaplan-Meier method. Even though the 5 thiopurine-intolerant cases were excluded from the Tac group, the relapse-free survival rate was still significantly higher in the IFX group than in the Tac group. Previously, a few studies have reported the differences in long-term outcomes between IFX and CsA treatment [18–20]. Croft et al. recently reported that IFX was superior to CsA in terms of colectomy-free rates at 12 months [18]. Naves et al. reported that salvage treatment was more often required with CsA strategies than IFX strategies over 3 years of follow-up [20]. The present study that compared the efficacy of IFX and Tac, which is also categorized as a calcineurin inhibitor like CsA, revealed that the IFX-based strategy was superior to the Tac-based strategy.

Here, it is quite important to discuss the risk factors that lead to relapse during the IFX-based strategy and Tac-based strategy. In the IFX-based strategy, disease duration at treatment was identified as an independent risk factor for relapse. The patients with disease duration of less than 5 years had a significantly higher risk for relapse. Neither concomitant use of thiopurine nor withdrawal of IFX was related to relapse. Over the recent years, combination therapy with IFX and azathioprine has shown to be superior to IFX monotherapy in both short-term [27] and long-term [28] regimens. We confirmed that the concomitant use of thiopurine was not a significant factor for short-term response in IFX group (data was not shown). Furthermore, the results of Cox proportional hazard model showed that concomitant use of thiopurine was not related to relapse in long-term independently. We considered that the discrepancy between our results and those reported by the previous study was mainly derived from selection bias and the small number of patients recruited in our study. When treating patients with combination of IFX and thiopurine, we must focus on the risk for lymphomas. The risk for T-cell non-Hodgkin's lymphoma is higher with the use of TNF-*α* inhibitors in combination with thiopurines, but not with TNF-*α* inhibitors alone [29]. Therefore, further investigation is necessary to determine whether the combination therapy with IFX and thiopurines can be regarded as standard therapy for steroid-refractory UC. The appropriateness of withdrawal of IFX in a long-term remission state also remains controversial. Our results indicated that, in some cases, IFX could be successfully discontinued when the patients are in the remission state. Further investigation is also necessary to clarify this issue.

In the Tac-based strategy, the absence of concomitant use of thiopurines and discontinuing the use of Tac beyond 3 months were identified as independent risk factors for relapse. As described above, in the IFX group, concomitant thiopurine use was not related to relapse independently in the IFX group. However, the absence of concomitant use of thiopurine was significantly related to relapse in the Tac group. Here, it is quite important to recognize that the role of thiopurine for each treatment strategy is different. In the IFX group, thiopurine was administrated to intensify the efficacy of IFX. On the other hand, in the Tac group, thiopurine mainly plays a role in the maintenance treatment after withdrawal of Tac. We suppose that the mechanisms underlying the efficacy in thiopurine treatment might be different between thiopurine monotherapy and combination treatment with IFX. When initiating thiopurine monotherapy as maintenance treatment, upfront administration should be needed considering a lag time of action. On the other hand, in combination treatment with IFX, thiopurine might have different additional mechanisms of action, such as inhibiting antibody production to IFX, which could quickly work from the initiation of drug administration. Therefore, we suggest that initiating thiopurine at the same time with IFX should be adequate to intensify the short-term efficacy of IFX in combination therapy. Thus, we must comprehend that the results of efficacies of thiopurine administrations in the IFX and Tac treatment should be interpreted independently and should not be compared directly, because the role of thiopurine is different. In most of our patients in the Tac group, Tac treatment was generally withdrawn at 3 months after the first administration if clinical remission was achieved; the patients were subsequently transferred to thiopurine monotherapy. The results of the Kaplan-Meier curve analysis revealed that relapses frequently occurred especially after the withdrawal of Tac at 3 months. All 4 patients who continued Tac beyond 3 months did not experience relapse during the observation periods. According to these results, induction treatment using Tac up to 3 months followed by thiopurine monotherapy is not sufficient to maintain long-term remission in patients with steroid-refractory UC. In many cases, administration of Tac beyond 3 months might be needed to sustain long-term remission, but the efficacy of Tac as long-term maintenance treatment is not well established yet. Thus far, there have been only a few reports describing the efficacy and safety of long-term maintenance therapy using Tac [16, 24, 25]. Moreover, long-term Tac administration is known to cause chronic renal dysfunction [30]. Therefore, it is necessary for clinicians to clarify the efficacy and safety of long-term use of Tac as maintenance therapy for patients with steroid-refractory UC.

When considering the relapse after stopping Tac, we should also focus on the achievement of endoscopic remission before the drug withdrawal. Recent studies have identified mucosal healing on endoscopy as a key prognostic parameter in the management of inflammatory bowel diseases [31]. However, according to the results of our study, relapse was observed in not a small number of the patients with endoscopic remission or mucosal healing. The results indicate that endoscopic remission and mucosal healing are not sufficient condition to keep remission after stopping Tac. In order to keep sustained remission in the Tac-thiopurine bridging treatment, much higher therapeutic goal might be needed, such as histological healing or functional healing.

As for the long-term colectomy-free survivals, there was no difference between the IFX group and Tac group. However, the result should be carefully interpreted. In the Kaplan-Meier analysis and log-rank test for colectomy-free survival, data collections were ended when the patients relapsed in order to evaluate the pure long-term efficacy of each treatment strategy. When relapses were observed, the patients were treated by changing the agents. If we collected the data even after the relapse, the colectomy-free survival would be strongly affected by the efficacies of newly switched drugs. As shown in Figure 4, the patients in the Tac group frequently relapsed and the observation periods in the Tac group were shorter than those in the IFX group (Table 1). Thus, we consider that the most important result of our study is the relapse-free survivals and the result of colectomy-free survival should only be regarded as a reference.

In this study, the frequency of adverse events seemed to be much higher in the Tac group than in the IFX group. However, most of the events observed in the Tac group were clinically mild and the frequency of the severe adverse events was similar in IFX group and Tac group. Therefore, the results suggest that clinicians might not focus much on the frequency of adverse events when selecting the appropriate treatment. Here, we must describe a limitation when interpreting the frequency of adverse events in this study. Although the present study was retrospectively designed, we could successfully collect precise data, such as renal function, liver function, and anemia because they were numeric data easily reviewed in medical records. On the other hand, as for the clinical symptoms such as tremor, there was a possibility that we failed to collect some events that were not described in the records. This is an important limitation when evaluating the adverse events in the study.

Limitations of our study include the selection bias, sample size, differences of the timing among the patients in evaluating the short-time efficacies, differences of the proportions of administrated thiopurine, and single-center experience. In particular, the most important limitations include the selection bias when choosing one of the regimens (IFX- or Tac-based strategy) and the imbalance in the baseline patient backgrounds in each group. In this study, criteria for choosing IFX or Tac depended on the treatment policy in our hospital. Basically, IFX was mainly chosen for thiopurine-refractory or thiopurine-intolerant patients, and Tac was chosen for thiopurine-naïve patients. Because the treatment period with Tac is limited up to 3 months, maintenance treatment with thiopurine is necessary after withdrawing Tac administration. Therefore, Tac treatment is not preferable for a thiopurine-refractory or thiopurine-intolerant patient. On the other hand, IFX is preferable for such patients because the long-term maintenance treatment with IFX is available. The same treatment strategy is recommended in the review article by Naganuma et al. [32] and many Japanese experts in IBD field follow the strategy. Eventually, the strategies described above affected differences in the proportions of thiopurine-naïve patients between the IFX and Tac groups. The difference in the proportions of steroids refraction or dependence between the two groups is also explainable by these strategies. Because steroids-dependent patients tended to be treated with thiopurine, the IFX group included many more steroid-dependent patients than the Tac group. As for the difference in baseline disease activity, patients in the Tac group exhibited the higher disease activities than those in the IFX group. We consider that the difference in baseline disease activity was also caused by differences in the proportions of steroids refraction or dependence. In general, disease activities were much higher in the steroid-refractory patients than in steroid-dependent patients. Therefore, the mean disease activity in the Tac group, which had higher number of steroid-refractory patients, was eventually higher than that in the IFX group. We realized that this selection bias and the differences in baseline patient backgrounds could influence the results of our study and that these are the main limitations of our study. Thus, in our opinion, the results of our study should not be interpreted as head-to-head comparisons of IFX and Tac. The results should rather be interpreted as the real treatment outcomes of IFX- or Tac-based treatment under certain selective treatment strategies. There are no comparison reports of IFX and Tac, and this is the first report in this field. Therefore, randomized controlled trial is necessary to directly compare the efficacy of these two drugs.

## 5. Conclusions

In conclusion, this report presents the findings of a single-center retrospective study of the relative efficacies of IFX- or Tac-based strategy for steroid-refractory UC. Our study showed that IFX and Tac have similar short-term therapeutic efficacy for the treatment of steroid-refractory UC. Maintenance treatment with IFX, however, yields better long-term outcomes than Tac-thiopurine bridging treatment. Because our data reflected the therapeutic outcomes under the certain selective treatment strategy, further randomized controlled trials are required to discern whether there is a clear discrepancy between the efficacies of these agents.

## Figures and Tables

**Table 1:** Baseline characteristics of patients in infliximab group and tacrolimus group.

	Infliximab group	Tacrolimus group	*p* value
	(*n* = 48)	(*n* = 47)	
Sex			0.93
Men (%)	31 (64.6)	30 (63.8)	
Female (%)	17 (35.4)	17 (36.2)	
Age at diagnosis (y)	24 (12–59)	30 (12–67)	0.041
Age at start of the treatment (y)	31 (14–67)	34 (15–70)	0.25
Disease duration at the treatment (y)	4 (0.6–32)	3 8 (0.1–18)	0.031
Disease extension			0.72
Left-sided colitis (%)	17 (35.4)	15 (31.9)	
Extensive colitis (%)	31 (64.6)	32 (68.1)	
Response to corticosteroids			0.003
Steroids refractory (%)	17 (35.4)	31 (66.0)	
Steroids dependence (%)	26 (54.2)	16 (34.0)	
Others (%)	5 (10.4)	0 (0)	
Thiopurine-naïve (%)	22 (45.8)	38 (80.9)	0.0004
Previous IFX treatment	—	5 (10.6)	—
Previous Tac treatment	14 (29.2)	—	—
Laboratory data at start of the treatment			
Hemoglobin (g/dL)	12.6 (8.6–17.5)	10.9 (7.2–15)	0.018
Albumin (g/dL)	3.8 (2.6–4.8)	3.1 (1.9–4.5)	<0.001
C-reactive protein (mg/dL)	0.4 (0.1–23.1)	1 (0.1–7.5)	0.01
Disease activity index at start of the treatment			
CAI	7 (2–19)	9 (4–16)	0.006
EI	8 (5–12)	8 (3–12)	0.005

Data are presented as median (range) or number (%).

CAI, Clinical Activity Index; EI, Endoscopic Index.

**Table 2:** Cox proportional hazard model for relapse in IFX group.

Parameter	Multivariate analysis (Cox proportional hazard model)		
	HR	95% CI	*p* value
Sex			0.65
Male	1		
Female	0.69	0.097–3.28	
Age at start of the treatment (y)			0.23
≥20	1		
<20	1.64 × 10^−9^	0–3.89	
Disease duration at the treatment (y)			**0.012**
≥5	1		
<5	9.17	1.54–181.6	
Disease Extension			0.33
Left-sided colitis	1		
Extensive colitis	2.69	0.42–52.1	
Response to corticosteroids			
Dependence	1		
Refractory	0.89	0.11–5.81	0.91
Others	9.01 × 10^−9^	0–34.7	0.56
Concomitant use of thiopurine			0.74
Yes	1		
No	1.36	0.21–10.6	
Withdrawal of infliximab			0.2
No	1		
Yes	2.07 × 10^−9^	1.15 × 10^−26^–4.00	

HR, hazard ratio; CI, confidence interval.

**Table 3:** Cox proportional hazard model for relapse in Tac group.

Parameter	Multivariate analysis (Cox proportional hazard model)		
	HR	95% CI	*p* value
Sex			0.79
Male	1		
Female	1.14	0.41–3.25	
Age at start of the treatment (y)			0.058
≥20	1		
<20	11.6	0.92–156.1	
Disease duration at the treatment (y)			0.68
≥5	1		
<5	1.35	0.33–6.54	
Disease extension			0.59
Left-sided colitis	1		
Extensive colitis	1.34	0.47–4.38	
Response to corticosteroids			0.97
Dependence	1		
Refractory	0.98	0.17–4.48	
Concomitant use of thiopurine			**0.027**
Yes	1		
No	6.83	1.27–32.4	
Continuing use of Tac beyond 3 months			**0.018**
Yes	1		
No	8.33 × 10^8^	1.56–1.8 × 10^290^	

HR, hazard ratio; CI, confidence interval.

**Table 4:** Adverse events during the treatments.

Adverse events (AEs)	Number of the patients	Serious AEs resulting in drug withdrawal
Infliximab group (*n* = 48)		
Infusion reaction	1	1
Delayed hypersensitivity reaction	1	1
Leukocytopenia	1	1
Anemia	2	0
Pneumonia	2	2
Idiopathic thrombocytopenic purpura	1	1
Total	**8**	**6 (12.5%)**

Tacrolimus group (*n* = 47)		
Renal dysfunction	8	1
Hot flushes	4	0
Tremor	4	0
Headache	5	0
Hypomagnesemia	5	0
Liver dysfunction	1	1
Total	**27**	**2 (4.3%)**
